# Rapid genomic characterization of SARS-CoV-2 viruses from clinical specimens using nanopore sequencing

**DOI:** 10.1038/s41598-020-74656-y

**Published:** 2020-10-15

**Authors:** Jun Li, Haoqiu Wang, Lingfeng Mao, Hua Yu, Xinfen Yu, Zhou Sun, Xin Qian, Shi Cheng, Shuchang Chen, Junfang Chen, Jingcao Pan, Jueliang Shi, Xuchu Wang

**Affiliations:** 1 Microbiology Laboratory, Hangzhou Center for Disease Control and Prevention, No. 568 Mingshi Road, Jianggan District, Hangzhou City, 310021 Zhejiang China; 2 Hangzhou Baocheng Biotechnology Co., Ltd., Hangzhou City, 310052 Zhejiang China; 3 Department of Infectious Diseases, Hangzhou Center for Disease Control and Prevention, Hangzhou City, 310021 Zhejiang China

**Keywords:** Next-generation sequencing, Genetic variation, Viral infection

## Abstract

The novel SARS-CoV-2 outbreak has swiftly spread worldwide. The rapid genome sequencing of SARS-CoV-2 strains has become a helpful tool for better understanding the genomic characteristics and origin of the virus. To obtain virus whole-genome sequences directly from clinical specimens, we performed nanopore sequencing using a modified ARTIC protocol in a portable nanopore sequencer and validated a routine 8-h workflow and a 5-h rapid pipeline. We conducted some optimization to improve the genome sequencing workflow. The sensitivity of the workflow was also tested by serially diluting RNA from clinical samples. The optimized pipeline was finally applied to obtain the whole genomes of 29 clinical specimens collected in Hangzhou from January to March 2020. In the 29 obtained complete genomes of SARS-CoV-2, 33 variations were identified and analyzed. The genomic variations and phylogenetic analysis hinted at multiple sources and different transmission patterns during the COVID-19 epidemic in Hangzhou, China. In conclusion, the genomic characteristics and origin of the virus can be quickly determined by nanopore sequencing following our workflows.

## Introduction

In December 2019, an outbreak of atypical pneumonia with an unclear etiology began in Wuhan, a major transportation hub in the center of China^1^. A novel coronavirus similar to severe acute respiratory syndrome coronavirus (SARS-CoV) was identified as the causative pathogen^2^, which was officially named SARS-CoV-2 by the International Committee on Taxonomy of Viruses (ICTV). Most previously known human-coronaviruses (HCoV) only cause mild upper respiratory infections (HCoV-229E, HCoV-NL63, HCoV-OC43 and HCoV-HKU1)^3^, but HCoVs sometimes cross species boundaries and cause fatal respiratory disease and outbreaks, as observed in the case of SARS-CoV^4^ and Middle East respiratory syndrome coronavirus (MERS-CoV)^5^. The seventh HCoV, SARS-CoV-2, underwent a spillover event in late December 2019 and then swiftly spread across the borders of cities and provinces in mainland China and soon became an emergency of major international concern. As of 9 July 2020, the cumulative number of confirmed human infections had increased to 11,841,326 (544,739 deaths), as reported by WHO (https://who.sprinklr.com/), which is almost 1500 times the total number of recorded SARS-CoV infections.

Hangzhou is a national tourism city with a registered population of 10.36 million located in the southern wing of the Yangtze River Delta, with a humid, subtropical climate facilitating the airborne survival and transmission of viruses associated with respiratory infections. The first case of COVID-19 was recorded in a returnee from Wuhan diagnosed on January 19, 2020. As of March 2020, 186 infections had been confirmed by viral RNA detection. As the virus genome can be sequenced rapidly in a portable MinION sequencer, the accurate genomic sequencing data that are generated could be used backward tracing to the origin during virus spreading, which could bring molecular epidemiology analysis close to the aim of front-line application.

Therefore, we applied a modified ARTIC protocol for SARS-CoV-2 genome sequencing on the MinION platform. Two workflows were applied and validated by amplifying and sequencing the genomes from the clinical samples of SARS-CoV-2-infected patients, and the characteristics of 29 SARS-CoV-2 genomes collected in Hangzhou were analyzed to study the origin and transmission history of these viruses.

## Results

### 8 h and 5 h workflows for SARS-CoV-2 nanopore sequencing

**Figure 1:**
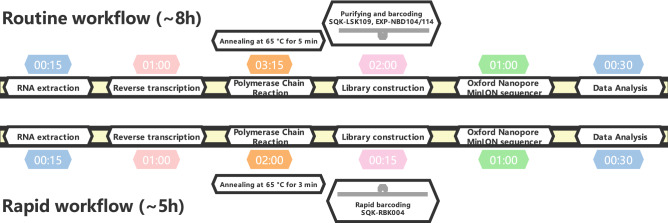
Overview of two nanopore sequencing workflows drawn by an online website (https://www.processon.com/). The white boxes represent the series of tasks that are the components of the 8-h routine workflow and the 5-h rapid workflow. The numbers in the colored boxes indicate the time required to complete the tasks.

**Figure 2:**
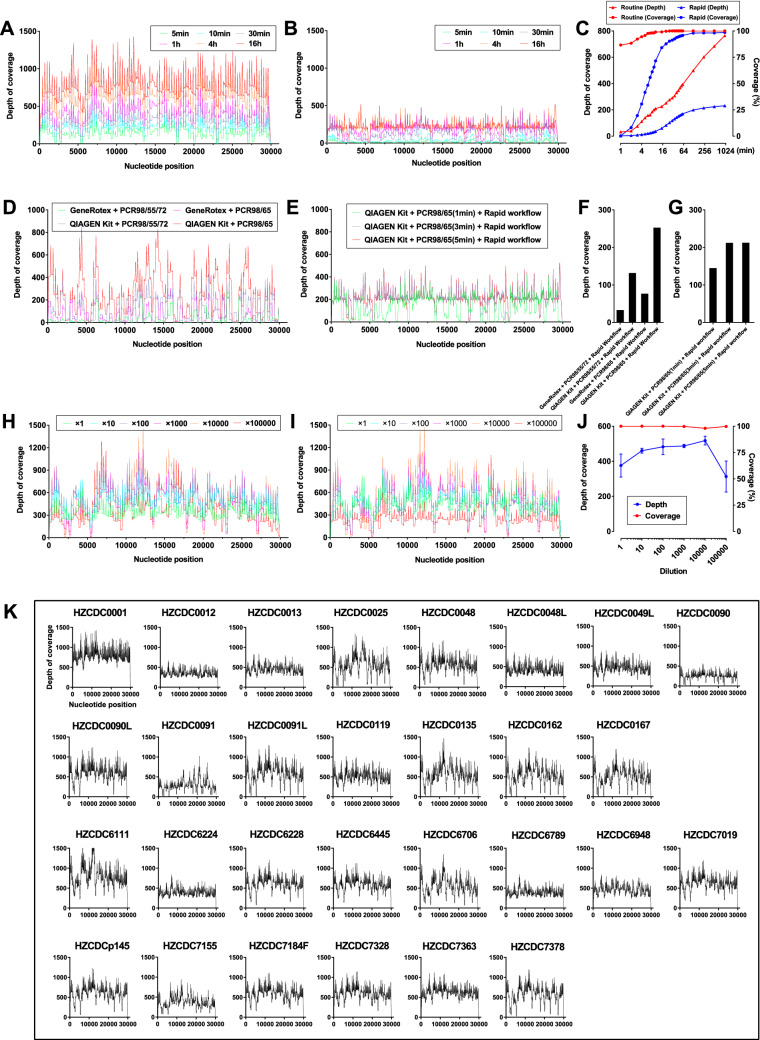
Analysis of the genome sequencing data for the SARS-CoV-2 viruses obtained with Oxford Nanopore Technologies using a MinION sequencer. (**A**) Trends of the depth data appearing over 16 h of sequencing using a Ligation Sequencing Kit 109. (**B**) The genome-wide depth of coverage using the Rapid Barcoding Kit 004. (**C**) Comparative analysis of the average depth and genome-wide coverage between the routine workflow and the rapid workflow. (**D**–**G**) Method optimization for nucleic acid extraction (magnetic bead or spin column method) and PCR amplification. (**H**–**J**) Repeated nanopore sequencing assays of viral RNA at a tenfold dilution. (**K**) The depth of genome-wide coverage appearing when the optimized methods were applied to 29 clinical specimens.

To acquire the whole-genome sequence of SARS-CoV-2 more efficiently, an 8-h workflow was designed on the basis of the sequencing throughput and speed after loading the library into the flow cell, and a 5-h workflow was designed for rapid library building (Fig. 1). These two workflows were both tested on the HZCDC0001 sample, with Ct values of 26.51/27.03 (Orf1ab/N); this sample was obtained from the first case that appeared in Hangzhou, Zhejiang Province. In the 8 h workflow, a nanopore ligation sequencing kit was applied, since this protocol can maximize the sequencing throughput and the length of reads (Fig. 2C). The total bases sequenced and the genome completeness of SARS-CoV-2 increased much faster than in the 5 h workflow. Here, regions with a depth greater than 15 × are recognized as the credible coverage, and genome completeness can reach almost 100% in only 10 min after loading the library into the flow cell. In contrast, the 5 h workflow took more than 1.5 h to approach 100% completeness. The 5 h workflow presented the advantage of a rapid 15-min library preparation time, especially under extreme conditions. This workflow greatly shortened the library preparation time compared to the 2-h ligation protocol (Fig. 1). However, as the rapid nanopore protocol cleaves the DNA to quickly add transposase adapters, the sequencing throughput and speed were poor compared with those of the 8 h workflow (Fig. 2A–C). In both workflows, two regions (5231–5644 bp and 22,798–23,214 bp, primer pairs #18 and #76) appeared to be short boards in genome mapping, which indicates a need for further optimization.

We performed some optimization to improve the workflow. Since RNA extraction is vital for follow-up sequencing, we compared magnetic bead extraction in an NP968 instrument (Tianlong, China) with column RNA extraction using the RNeasy Mini Kit (QIAGEN, Germany) (Fig. 2D). The latter approach seemed to yield higher-quality RNA, nearly doubling the depth of coverage (Fig. 2F). Moreover, the PCR procedure took more than 40% of the total workflow time, so we tried to decrease the annealing and extension time from 5 to 3 min and 1 min, corresponding to a total time during PCR of approximately 3–1 h. Even when the annealing time was reduced to 1 min, the whole-genome sequence could still be obtained from the products of the 1 h PCR procedure (Fig. 2E,G). Because of the different viral titers of SARS-CoV-2 in the clinical samples, the 3-min annealing time could be considered the equilibrium point.

### Sensitivity test and application in clinical samples

As clinical specimens may exhibit extremely low viral titers, we tested the sensitivity of the routine 8-h workflow by serially diluting RNA from two COVID-19 patients 10 times (starting from RNA samples with a Ct value ~ 22) to determine whether we could amplify the whole genome of SARS-CoV-2 from samples that showed failure in the qRT-PCR test. Approximately 85% of the reads were mapped to the reference genome (GenBank accession number MN908947.3) with an average depth of coverage greater than 250 × across > 97.56% of the SARS-CoV-2 genome for both samples, including the 100,000 ×-diluted sample, which was undetectable by the qRT-PCR test in an ABI 7500 instrument (Fig. 2H–J). The average depth was not obviously decreased among the diluted samples; however, the genome-wide depth fluctuated very significantly when the dilution rate was above 10,000 ×.

The routine workflow was applied to obtain the genomes from 29 clinical samples with an average depth of 233.75–754.88 × and genome completeness of 98.08–100% (Table 1). The information for the specimens, including the genome-wide depth of coverage determined by nanopore sequencing and Ct values determined by RT-PCR, is listed in Table 1 and Fig. 2K. In particular, the genomes of some specimens with low Ct values, such as HZCDC0090 and HZCDC0091, were also obtained with completeness ranging from 99.15 to 100%, which provided evidence that some problematic clinical samples can be successfully sequenced via nanopore sequencing. In addition, both upper and lower respiratory tract specimens (HZCDC0048, HZCDC0048L, HZCDC0090, HZCDC0090L, HZCDC0091 and HZCDC0091L) from three COVID-19 patients were sequenced, and the genome mapping results showed that the virus genomes from different parts of the respiratory tract were consistent (Table 1).

**Table 1:** Information for 29 Hangzhou SARS-CoV-2 viruses from COVID-19 patients in this study.

Strain number	Source	Gender	Age	Date of onset	Collection date	History	qRT-PCR (Ct)		GISAID accession number	Average depth	Completeness (%)
							ORF1a/b	N			
HZCDC0001	Sputum	Male	31	2020-01-18	2020-01-19	Infected in Wuhan	26.51	27.03	EPI_ISL_407313	754.88	100.00
HZCDC0012	Nasal, oropharyngeal swab	Male	47	2020-01-18	2020-01-20	Spouse of HZCDC0013	22.2	21.67	EPI_ISL_421236	326.64	100.00
HZCDC0013	Nasal, oropharyngeal swab	Female	45	2020-01-15	2020-01-20	Infected in Wuhan	22.34	21.88	EPI_ISL_421235	418.66	100.00
HZCDC0025	Nasal, oropharyngeal swab	Male	51	2020-01-21	2020-01-21	Infected in Wuhan	35.42	34.83	EPI_ISL_421234	509.46	99.99
HZCDC0048	Nasal, oropharyngeal swab	Male	35	2020-01-16	2020-01-21	Contact with a patient from Wuhan	27.95	27.72	EPI_ISL_421233	506.14	100.00
HZCDC0048L	Tracheal aspirate sample						19.7	19.58	EPI_ISL_421232	385.17	100.00
HZCDC0049L	Tracheal aspirate sample	Female	40	2020-01-17	2020-01-21	Contact with a patient from Wuhan	25.11	24.64	EPI_ISL_421231	413.09	100.00
HZCDC0090	Nasal, oropharyngeal swab	Female	34	2020-01-17	2020-01-21	Contact with a patient from Wuhan	Neg	Neg	EPI_ISL_421230	233.75	99.15
HZCDC0090L	Bronchoalveolar-lavage fluid						30.29	29.54	EPI_ISL_421229	555.60	100.00
HZCDC0091	Nasal, oropharyngeal swab	Male	31	2020-01-17	2020-01-21	Contact with a patient from Wuhan	Neg	37.14	EPI_ISL_421228	264.45	99.16
HZCDC0091L	Bronchoalveolar-lavage fluid						33.09	32.95	EPI_ISL_421227	535.46	99.97
HZCDC0119	Nasal, oropharyngeal swab	Female	41	2020-01-21	2020-01-22	Contact with a patient from Wuhan	26.2	25.99	EPI_ISL_421226	464.11	100.00
HZCDC0135	Nasal, oropharyngeal swab	Male	62	2020-01-15	2020-01-22	Infected in Wuhan	35.5	34.79	EPI_ISL_421225	509.26	99.95
HZCDC0162	Nasal, oropharyngeal swab	Male	46	2020-01-21	2020-01-23	–	35.93	34.96	EPI_ISL_421224	488.18	99.61
HZCDC0167	Nasal, oropharyngeal swab	Female	53	2020-01-21	2020-01-23	–	33.33	32.85	EPI_ISL_421223	464.55	98.08
HZCDC6111	Nasal, oropharyngeal swab	Female	39	2020-03-04	2020-03-05	Contact with imported cases	28.51	28.53	EPI_ISL_482575	576.70	100.00
HZCDC6224	Nasal, oropharyngeal swab	Female	39	2020-03-04	2020-03-06	Contact with imported cases	29.47	28.79	EPI_ISL_482576	558.21	100.00
HZCDC6228	Nasal, oropharyngeal swab	Male	29	2020-03-06	2020-03-07	Infected in France	31.74	31.17	EPI_ISL_482577	459.86	100.00
HZCDC6445	Nasal, oropharyngeal swab	Male	46	2020-03-08	2020-03-12	Contact with imported cases	26.97	27.67	EPI_ISL_482578	550.81	100.00
HZCDC6706	Nasal, oropharyngeal swab	Female	37	2020-03-12	2020-03-14	Infected in U.S.A	26.71	28.10	EPI_ISL_421222	507.37	100.00
HZCDC6789	Nasal, oropharyngeal swab	Male	21	2020-03-13	2020-03-15	Infected in U.K	22.79	22.41	EPI_ISL_421221	365.88	100.00
HZCDC6948	Nasal, oropharyngeal swab	Male	17	2020-03-16	2020-03-17	Infected in U.K	17.79	18.29	EPI_ISL_482579	434.33	100.00
HZCDC7019	Nasal, oropharyngeal swab	Female	43	2020-03-17	2020-03-18	Mother of HZCDC6948	26.12	26.41	EPI_ISL_482580	588.17	100.00
HZCDCp145	Nasal, oropharyngeal swab	Male	12	2020-03-19	2020-03-21	Infected in Switzerland	33.99	36.89	EPI_ISL_482581	577.25	100.00
HZCDC7155	Nasal, oropharyngeal swab	Female	26	2020-03-19	2020-03-21	Infected in Switzerland	24.44	25.82	EPI_ISL_482582	351.02	100.00
HZCDC7184F	Feces	Male	23	2020-03-07	2020-03-22	Contact with imported cases	19.83	20.89	EPI_ISL_482583	560.85	100.00
HZCDC7328	Nasal, oropharyngeal swab	Female	21	2020-03-22	2020-03-24	Infected in France	23.02	25.03	EPI_ISL_482584	562.57	100.00
HZCDC7363	Nasal, oropharyngeal swab	Male	21	2020-03-24	2020-03-25	Infected in France	17.77	17.19	EPI_ISL_482585	590.53	100.00
HZCDC7378	Nasal, oropharyngeal swab	Female	15	2020-03-25	2020-03-25	Infected in U.S.A	29.11	28.74	EPI_ISL_482586	541.30	100.00

### Genomic variations and phylogenetic analyses of SARS-CoV-2

The length of the reference SARS-CoV-2 genome (MN908947.3) was 29,903 bp. However, a considerable fraction of the submitted SARS-CoV-2 genomes were incomplete. Therefore, the strategy of building a phylogenetic tree based on SNPs was applied to investigate the traceability of samples of interest. To avoid introducing errors in nanopore sequencing, we first filtered low-quality reads, and only SNPs with high quality (Phred value ≥ 20) and a high site depth of coverage (≥ 50) were considered in the downstream analysis. In addition, we performed Illumina sequencing for all 29 clinical samples, which provided evidence that the SNPs from our standard were 100% consistent with the Illumina data and that SNPs from SARS-CoV-2 genomes can be called on the basis of nanopore sequencing alone.

**Figure 3:**
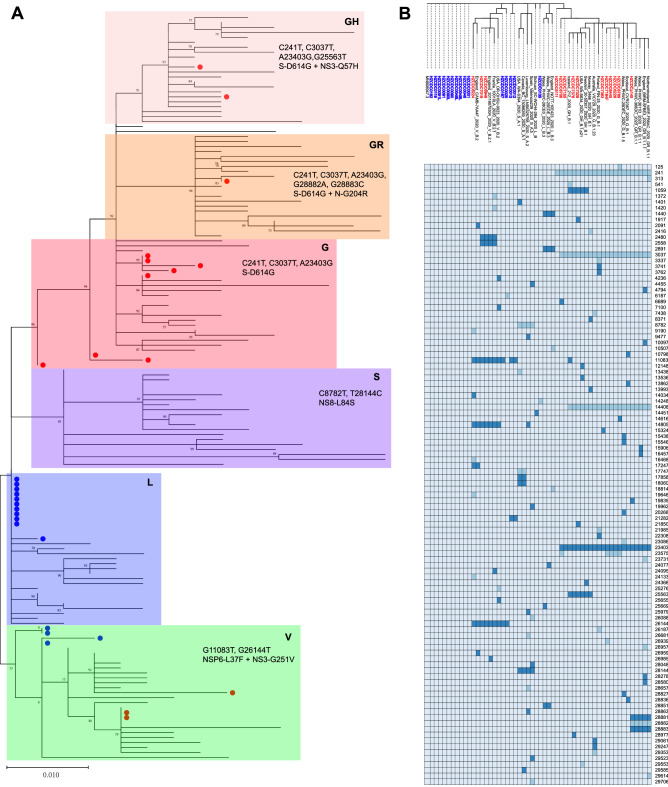
Phylogenetic analysis of 29 Hangzhou SARS-CoV-2 strains with reference genome sequences. (**A**) The phylogenetic relationships of the 29 Hangzhou SARS-CoV-2 genomes with 196 reference sequences from GISAID. The genomes collected in January (blue) or March (red) are indicated by solid circles with different colors, and the genetic clusters are differentiated according to which clade they belong to. Signature nucleotide substitutions and amino acid changes are annotated with their positions. (**B**) The condensed phylogenetic tree with the matrix of variations plotted in a heat map drawn with phyD3 version 1.3 (https://phyd3.bits.vib.be/). The missense and synonymous variants are indicated with dark blue and light blue, respectively. The numbers listed on the side are genome positions according to the reference SARS-CoV-2 genome (GenBank accession number MN908947).

In all 29 obtained complete SARS-CoV-2 genomes, 33 substitutions distributed in five coding sequences (CDSs) and 5′UTRs were identified based on sequence alignment (Fig. 3), including C125T and C241T in the 5′UTR, 10 synonymous variations and 21 missense variations (Table 2). Among the 21 amino acid variants, two were found in the S gene (E96D and D614G), three were found in the N gene (R203K, G204R and S235F), and the others were detected in ORF1a/b (T265I, T551I, I739V, P765S, A2142S, L3606F, M4590T, A4784V, T4847I, T5020I, V5661A, I6525T and D7006G) and ORF3a (Q57H and G251V), respectively. The D614G amino acid change in the spike protein was confirmed to be associated with greater infectivity^6^, which was caused by an A23403G nucleotide mutation compared with MN908947.3. In our study, it was found that G614 in the spike protein had become the dominant in the imported cases, increasing from 0% (15 genomes in January) to 71.4% (10 of 14 genomes in March), accompanied by C241T in the 5′UTR, the silent mutation C3037T and the missense variation C14408T, which results in a P323L amino acid change in RNA-dependent RNA polymerase (RdRp).

**Table 2:** The list of substitutions in all 29 obtained complete genomes of SARS-CoV-2.

Site	Variations	Variation type	Gene	Amino acid change	Frequency
241	C → T	Intergenic region	5′UTR	None	1 (3.45%)
241	C → T	Intergenic region	5′UTR	None	11 (37.93)
1059	C → T	Missense variant	orf1ab	Thr265Ile	2 (6.90%)
1917	C → T	Missense variant	orf1ab	Thr551Ile	1 (3.45%)
2480	A → G	Missense variant	orf1ab	Ile739Val	2 (6.90%)
2558	C → T	Missense variant	orf1ab	Pro765Ser	2 (6.90%)
3037	C → T	Synonymous variant	orf1ab	None	10 (34.48%)
6187	C → T	Synonymous variant	orf1ab	None	1 (3.45%)
6689	G → T	Missense variant	orf1ab	Ala2142Ser	1 (3.45%)
9190	G → T	Synonymous variant	orf1ab	None	1 (3.45%)
11,083	G → T	Missense variant	orf1ab	Leu3606Phe	6 (20.69%)
14,034	T → C	Missense variant	orf1ab	Met4590Thr	1 (3.45%)
14,248	T → C	Synonymous variant	orf1ab	None	1 (3.45%)
14,408	C → T	Missense variant	RdRp	Pro323Leu	8 (27.59)
14,616	C → T	Missense variant	orf1ab	Ala4784Val	1 (3.45%)
14,805	C → T	Missense variant	orf1ab	Thr4847Ile	3 (10.34%)
15,324	C → T	Missense variant	orf1ab	Thr5020Ile	1 (3.45%)
16,468	C → T	Synonymous variant	orf1ab	None	1 (3.45%)
17,247	T → C	Missense variant	orf1ab	Val5661Ala	1 (3.45%)
19,646	T → C	Synonymous variant	orf1ab	None	1 (3.45%)
19,839	T → C	Missense variant	orf1ab	Ile6525Thr	1 (3.45%)
21,282	A → G	Missense variant	orf1ab	Asp7006Gly	2 (6.90%)
21,850	G → T	Missense variant	S	Glu96Asp	1 (3.45%)
23,403	A → G	Missense variant	S	Asp614Gly	10 (34.48%)
23,575	C → T	Synonymous variant	S	None	5 (17.24%)
24,133	C → T	Synonymous variant	S	None	1 (3.45%)
25,563	G → T	Missense variant	ORF3a	Gln57His	2 (6.90%)
26,144	G → T	Missense variant	ORF3a	Gly251Val	5 (17.24%)
26,939	A → G	Synonymous variant	M	None	1 (3.45%)
28,881	G → A	Missense variant	N	Arg203Lys	1 (3.45%)
28,882	G → A	Synonymous variant	N	None	1 (3.45%)
28,883	G → C	Missense variant	N	Gly204Arg	1 (3.45%)
28,977	C → T	Missense variant	N	Ser235Phe	1 (3.45%)

Two family clusters of SARS-CoV-2 infections involving 4 patients were found. One of the index patients (HZCDC0013) was a female who was infected in Wuhan before returning home and was diagnosed with COVID-19 five days later. Her husband (HZCDC0012) was confirmed to have SARS-CoV-2 infection on the same day, with a strain showing with two mutations, G11083T and A21282G, consistent with HZCDC0013. In another human-to-human transmission event, identical substitutions (A2480G, C2558T, G11083T, C14805T and G26144T) were found in the genomes obtained from a mother and a son, hinting at a family-cluster transmission history of SARS-CoV-2 from a U.K. student to his mother.

The phylogenetic analysis was conducted based on a July 4, 2020 download of 196 SARS-CoV-2 reference genomes from different lineages^7^ and clades from the GISAID database (GISAID acknowledgments are included in Table S1) randomly selected by the Perl rand() function. According to the observed genetic diversity, four clades, L, V, S and G, were defined, among which the L clade was the most prominent among the genomes collected in January 2020 except four of fifteen genomes belonging to clade V showing an L3603F substitution in ORF1a/b and a G251V substitution in ORF3a (Fig. 3). Clade V strains were also found in three samples collected from imported cases in March 2020. However, most of the genomes from the patients who had a history of going abroad or of contact with imported confirmed cases were included in the G clade, which harbors the D614G substitution. The G clade is currently the most prominent clade and has been additionally subdivided into the G, GR and GH subclades. The phylogenetic relationships revealed that most of our genomes from COVID-19 patients linked to imported cases were scattered among the three subclades, with 8 cases in G, 1 case in GR, and 2 cases in GH (Fig. 3), which hinted at multiple sources of transmission from overseas.

## Discussion

The outbreak of COVID-19 caused by SARS-CoV-2 has swiftly spread worldwide. However, the probable origin of SARS-CoV-2 associated with the COVID-19 pandemic is still unclear^8^. Recent reports of COVID-19 cases with no or mild upper respiratory tract symptoms suggest the potential for asymptomatic or oligosymptomatic transmission during the first week of symptoms^9,10^. Hence, there is an urgent need for rapid identification and traceability of pathogens for disease control and prevention. A deep understanding of the novel virus is first obtained through the analysis of the genome sequence. In this study, we demonstrated the utility of nanopore sequencing for SARS-CoV-2 genomes from clinical specimens based on a modified ARTIC protocol. The adopted approach allowed the confirmation of SARS-CoV-2 infections at the genomic level within a few minutes by sequencing and simultaneously mapping the reads to the reference genome and analyzing the output data in real time.

Compared with nasal/oropharyngeal swabs, the virus could be detected more readily in lower respiratory tract specimens from COVID-19 patients^11^. Our data showed that the virus genomes from different parts of the respiratory tract were consistent. However, the difference in viral loads among samples will affect the stability of the average depth and genome-wide coverage and increase the difficulty of whole-genome mapping, suggesting the importance of sample collection for later genome sequencing.

To characterize the genomic variations, we found 33 different substitution sites distributed in five coding regions among 29 SARS-CoV-2 genomes, without any recombination events. Genomic evidence supported the linkage of most of the early 15 infections with Wuhan either directly or indirectly, and 10 of the 14 genomes from the imported infections that occurred in March 2020 exhibited the specific D614G variation in the spike protein that belongs to clade G compared with the domestic strains.

The small sample size of 29 clinical samples is a significant limitation of this study. Although the number of genomes was not large, the typical enrolled cases were representative. First, this cohort included various forms of transmission, including local cases, domestic spreading from a Wuhan returnee, transmission from imported cases from multiple countries, familial cluster infections (HZCDC0012 and HZCDC0013, HZCDC6948 and HZCDC7019), and a small-scale outbreak (HZCDC0048, HZCDC0049, HZCDC0090, HZCDC0091 and HZCDC0119). Second, the mean (± SD) age of the patients was 34.81 ± 12.79 years and was distributed between 12 and 62 years old, including 53.85% males and 46.15% females. Moreover, five different types of samples were used to test the approach of SARS-CoV-2 sequencing based on nanopore technology, including nasal and oropharyngeal swabs, sputum, tracheal aspirates, bronchoalveolar lavage fluid from the respiratory tract and fecal specimens from the digestive tract, showing a wide range of applications in different types of clinical samples.

In summary, we performed SARS-CoV-2 genome sequencing in a portable nanopore sequencer. Combined with the 8-h workflow, the genomic characteristics and the origin of the virus could be quickly determined. The rapid 5-h workflow, with 15-min fast library preparation could be applied for backward tracing of the strains out of lab, bringing genome-level molecular epidemiology analysis to the front lines of the outbreak. Therefore, based on prompt diagnosis and rapid whole-genome analysis, the swift and decisive response to the SARS-CoV-2 outbreak will benefit disease control and prevention efforts.

## Methods

### Ethics statement

This study and all experimental protocols performed were approved by the Institutional Review Board of the Hangzhou Center for Disease Control and Prevention. We confirm that all methods were carried out in accordance with relevant guidelines and regulations. Signed informed consent was obtained from the patients or their spouses, or the parents of minor*,* and personal identification information was anonymized.

### Viral infection diagnosis

Upper and/or lower respiratory tract samples, including nasal and oropharyngeal swabs, sputum, tracheal aspirates and bronchoalveolar lavage fluid, and fecal specimens from the digestive tract, were collected from suspected cases with informed consent from the patients or their spouses and were sent on ice to the Hangzhou Center for Disease Control and Prevention for diagnosis within six hours. The viral RNA was extracted directly from 200 μL of the supernatant of the clinical samples using the RNeasy Mini Kit (QIAGEN, Germany) according to the manufacturer’s instructions in a biosafety cabinet in Biosafety Level 2 Laboratory and tested for the presence of SARS-CoV-2 using the diagnostic real-time reverse transcription polymerase chain reaction (qRT-PCR) test on an ABI7500 instrument (ABI, USA) following the diagnostic kit manual.

### Workflows of virus genome sequencing

Viral RNA extracted from clinical samples was used as a template to amplify and sequence the SARS-CoV-2 genome. Briefly, cDNA was synthesized from 11 μL of viral RNA using the SuperScript IV First-Strand Synthesis System (Invitrogen, USA) with random hexamers. PCR was performed using Q5 Hot Start High-Fidelity DNA Polymerase (NEB, USA) and a set of primers targeting regions of the SARS-CoV-2 genome designed by the ARTIC network (https://artic.network/ncov-2019). The PCR mixture was initially incubated for 2 min at 98 °C for denaturation, followed by 35 cycles of 98 °C for 15 s and 65 °C for 1, 3 or 5 min (depending on the workflows). The amplified products were purified with an equal volume of AMPure XP beads (Beckman Coulter, USA) to exclude small nonspecific fragments.

According to the eight-hour routine workflow (Fig. 1), the purified DNA was repaired with NEBNext FFPE Repair Mix (NEB, USA), followed by DNA end preparation using NEBNext End repair/dA-tailing Module (NEB, USA) and the successive attachment of native barcodes and sequencing adapters supplied in the EXP-NBD104/114 kit (Oxford Nanopore Technologies, UK) to the DNA ends. The DNA concentration was determined with a Qubit 3.0 instrument using a dsDNA HS Assay Kit (Thermo Fisher, USA). After priming the flow cell, 60 ng of DNA per sample of the products was pooled in a DNA library with a final volume of 65 μL. Following the ligation sequencing kit (SQK-LSK109, Oxford Nanopore Technologies, UK) protocol, MinION Mk1B was used to perform genome sequencing in an R9.4.1 flow cell for 1 h per sample. For the rapid barcoding workflow, a fragmentation mixture from the SQK-RBK004 kit (Oxford Nanopore Technologies, UK) was used to attach the barcodes to the DNA ends, followed by the attachment of sequencing adapters.

### Read preprocessing and consensus building for nanopore sequencing

Base calling was performed by using guppy (https://community.nanoporetech.com) with the parameter settings “-c dna_r9.4.1_450bps_hac.cfg -x auto”, different samples were separated, and adapters were trimmed with the additional parameter settings “-trim_barcodes -barcodes EXP-NBD104/EXP-NBD114/SQK-RBK004”. FASTQ reads were filtered for quality control according to a cutoff “length ≥ 200 and Phred value ≥ 7” using the program “filtlong v0.2.0” (https://github.com/rrwick/Filtlong).

After the application of read quality control procedures, the artic-ncov2019 pipeline (https://artic.network/ncov-2019) was applied to perform sequence mapping, primer trimming, variant calling and consensus assembly building. Variations were called using Medaka 0.11.1 (https://github.com/nanoporetech/medaka). In the stage of consensus assembly building, sites with a depth lower than 50 × were masked by N bases, and the reference was substituted by homozygous variations with a Phred quality ≥ 20. “Samtools depth” was used to calculate the depth of each site, and “Samtools bedcov” was used to calculate the window depth for scanning in the genome^12^.

### Read preprocessing and variant calling for Illumina sequencing

Raw Illumina PE reads were trimmed and subjected to quality control with the software fastap 0.20.0 with default parameters^13^. Bwa 0.7.17-r1188^14^ was used to map the clean reads to the SARS-CoV-2 reference genome, and SAM/BAM files were manipulated by using SAMtools 1.9^12^. Variations were detected with the program “mpileup and calling” from bcftools 1.9^15^. Variations were considered positive when they exhibited a Phred quality value ≥ 20 and a depth ≥ 50.

### Phylogeny and variant analysis

To remove bias from the gaps in the incomplete genome, sequence alignment according to all SNP sites was chosen to build the phylogenetic tree. First, all SNPs were called with alignment to SARS-CoV-2 reference sequences using the “nucmer” and “dnadiff” programs from MUMmer 3.23^16^, and the effect of the SNPs was estimated using SnpEff 4.3t^17^. Second, all SNP sites were connected to a single sequence for every sample based on the variant calling results from the last step. Then, these sequences were combined to perform phylogenetic analysis, and maximum likelihood phylogenies were estimated by using FastTree 2.1.10^18^ with the default parameters. The phylogenetic tree with the variation heatmap matrix was drawn by using phyD3^19^. The group and clade numbers were assigned to achieve consistency with earlier studies.

## Supplementary information

Supplementary Table 1.

## Data Availability

All genome sequences included in this study are available from GISAID (https://gisaid.org) (the full list of the accession numbers is available in Table 1).
